# Predictors of histologically confirmed local recurrence and no evident association of wound soaker catheter use with local recurrence in dogs with grade II–III mammary carcinomas: a retrospective cohort study

**DOI:** 10.3389/fvets.2026.1772385

**Published:** 2026-03-27

**Authors:** Manuel Fuertes-Recuero, Guillermo Valdivia, María Suarez-Redondo, Paula García San José, Silvia Penelo, Mario Arenillas, Laura Peña, Dolores Pérez-Alenza, Gustavo Ortiz-Díez

**Affiliations:** 1 Complutense Veterinary Teaching Hospital, Complutense University of Madrid, Madrid, Spain; 2 Department of Animal Medicine and Surgery, Veterinary Medicine School, Complutense University of Madrid, Madrid, Spain

**Keywords:** canine mammary carcinoma, histologic grade, local recurrence, mastectomy, postoperative analgesia, risk factors, wound soaker catheter

## Abstract

**Introduction:**

Local recurrence after mammary surgery is a clinically relevant outcome in dogs with mammary carcinomas, yet recurrence-specific prognostic factors and the oncologic safety of wound soaker catheters (WSCs) remain insufficiently defined.

**Methods:**

This retrospective cohort study aimed (1) to identify clinicopathologic factors associated with time to histologically confirmed local recurrence in dogs with grade II–III mammary carcinomas and (2) to evaluate whether WSCs delivering intermittent bupivacaine boluses were associated with the hazard of local recurrence. Standardized records from a veterinary teaching hospital were reviewed for female dogs undergoing nodulectomy, regional mastectomy, or unilateral radical mastectomy with histologic confirmation of grade II–III carcinoma. Local recurrence was strictly defined as histologic regrowth at or near the surgical site, and time to local recurrence was analyzed using Cox proportional hazards models.

**Results:**

The cohort included 117 dogs (65.0% grade II; 35.0% grade III), of which 11 (9.4%) developed local recurrence, with a median time to recurrence of 6.4 months. In the multivariable model based on 107 complete cases, larger tumor size (hazard ratio [HR] 1.29 per 1-cm increase; 95% confidence interval [CI] 1.07-1.55) and histologic infiltration (HR 4.51; 95% CI 1.43-14.23) were independently associated with a higher hazard of local recurrence. WSC use was not associated with the hazard of local recurrence (HR 0.36; 95% CI 0.09–1.40), although the estimate was imprecise.

**Discussion:**

Overall, these findings suggest that tumor size and histologic infiltration are associated with histologically confirmed local recurrence, and they do not provide evidence that WSC-based local analgesia increases the hazard of local recurrence within the limitations of this retrospective cohort.

## 1 Introduction

Canine mammary carcinomas are among the most common malignancies in female dogs and remain a major clinical challenge in small-animal oncology (1–6). Prognosis is influenced by tumor size, clinical stage, lymph node status, and histologic type and grade (7–9). Histologic grade is a well-established prognostic factor, with higher grades consistently associated with poorer outcomes, including shorter survival and higher rates of recurrence and metastatic disease across retrospective (10, 11) and prospective cohorts (7, 8, 12). In addition, selected high-grade phenotypes, notably inflammatory mammary carcinoma, share clinicopathologic features with aggressive human breast cancer subtypes, supporting a One Health perspective in which some forms of canine mammary cancer are considered spontaneous comparative models (13–16).

Local recurrence after mastectomy is clinically relevant because it may require additional surgery, impair quality of life, and precede systemic progression. In canine mammary carcinoma cohorts, local recurrence is often embedded within composite endpoints (disease-free interval or locoregional relapse) that may also include new primary mammary tumors and/or distant metastasis, limiting recurrence-specific inference (10, 11). When evaluated as a discrete outcome, larger tumor size, higher clinical stage, ulceration, lymph vascular invasion, infiltrative growth patterns, and incomplete or close surgical margins have been associated with a higher likelihood of local recurrence (7–9). However, recurrence-specific predictors in dogs with histologic grade II–III mammary carcinomas treated under standardized protocols remain incompletely characterized.

Effective perioperative analgesia is essential for dogs undergoing mastectomy, a procedure associated with substantial postoperative pain (17–20). Wound soaker catheters (WSCs), which deliver local anesthetics along the surgical incision, are increasingly incorporated into multimodal analgesia protocols in small-animal practice. In veterinary patients, WSCs have been associated with effective postoperative analgesia, reduced opioid requirements, and low rates of minor complications such as seroma formation or catheter disconnection (21–25). Recent mastectomy studies have reported uneventful catheter management, no increase in surgical-site infection rates, and negative catheter cultures even without routine postoperative antibiotics (26, 27).

Despite these favorable perioperative outcomes, some surgeons remain cautious about WSC use in oncologic procedures. This concern reflects long-standing surgical oncology principles in which devices traversing a tumor bed are viewed as a potential route for tumor-cell implantation, a concept largely extrapolated from isolated reports of drain-tract recurrence in human breast cancer and expert opinion rather than veterinary clinical evidence (28–30). To date, no clinical study in dogs has evaluated whether WSC use is associated with the hazard of histologically confirmed local recurrence after mastectomy for mammary carcinoma. Preclinical studies suggest that local anesthetics, including bupivacaine, can exert antiproliferative or pro-apoptotic effects in tumor models, including canine mammary tumor cell lines, although clinical relevance remains uncertain (31, 32). In human oncology, observational studies and randomized trials comparing regional or local anesthetic techniques with conventional general anesthesia and systemic opioid analgesia have not described an increased risk of cancer recurrence overall (33–35). Although these findings cannot be directly extrapolated to dogs, they do not support a clinically meaningful oncologic harm signal from perioperative use of local anesthetics.

Therefore, we conducted a retrospective cohort study of female dogs with histologically confirmed grade II-III mammary carcinomas treated surgically at a single veterinary teaching hospital under a standardized institutional protocol. The primary objective was to identify clinicopathologic and clinical management factors associated with time to histologically confirmed local recurrence. The secondary objective was to evaluate whether WSC use for postoperative local bupivacaine delivery was associated with the hazard of local recurrence. Given the heterogeneous evidence for routine adjuvant systemic approaches and the scarcity of prospective randomized trials in canine mammary carcinoma, recurrence-specific estimates may also inform future study design (13). We hypothesized that indicators of tumor burden and local invasiveness would be associated with a higher hazard of histologically confirmed local recurrence after surgery, and that WSC based local anesthetic delivery would not be associated with an increased hazard of local recurrence.

## 2 Materials and methods

### 2.1 Study design, setting, and reporting

This retrospective cohort study was based on electronic medical records from the Complutense Veterinary Teaching Hospital (Madrid, Spain). The study population comprised female dogs with histologic grade II or III mammary carcinomas treated surgically between January 2012 and December 2024. Case management was standardized through the hospital Mammary Oncology Unit, which followed an institutional protocol for staging, surgical planning, histopathology reporting, perioperative analgesia, adjuvant therapy, and follow-up procedures. However, the uptake of specific perioperative analgesic techniques, particularly wound soaker catheter (WSC) placement, evolved over the study period (see Section 2.5), introducing potential calendar-time confounding for WSC-related analyses (26).

Reporting followed the Strengthening the Reporting of Observational Studies in Epidemiology (STROBE) recommendations for cohort studies (36). At hospital admission, owners signed a standard informed consent form authorizing anonymized use of clinical data, biological samples, and diagnostic images for teaching and research and permitting supervised participation of veterinary students in patient care. This study used only retrospective anonymized data generated during routine clinical management and, under institutional and university regulations, did not require additional project-specific ethical approval.

### 2.2 Eligibility criteria and study population

Dogs were eligible if they were female; underwent surgical excision of one or more mammary masses (nodulectomy, regional mastectomy, or unilateral radical mastectomy) during the study period; had a final histopathologic diagnosis of mammary carcinoma classified according to established canine mammary tumor histologic classification systems (37, 38) and graded as II or III using the Peña grading system (7); and had sufficient clinical and follow-up information to ascertain local recurrence status and follow-up time for time to event analysis.

Dogs were excluded if they had another concurrent malignant neoplasm at the time of surgery; evidence of distant metastatic disease (M1) on preoperative three-view thoracic radiography and abdominal ultrasonography using the modified WHO TNM staging framework (39); or clinical, surgical, or histopathologic records insufficient to reliably classify key variables or the outcome. To preserve outcome specificity, dogs with clinically suspected but not histologically confirmed local recurrence did not meet the pre-specified outcome definition and were excluded at cohort assembly. During the study period, 117 female dogs met all eligibility criteria and formed the analytic cohort. No matching or sampling procedures were applied; the study size was determined by the number of eligible cases identified in the hospital database.

### 2.3 Preoperative evaluation and clinical staging

Preoperative evaluation followed the Mammary Oncology Unit protocol (26). All dogs underwent a complete physical examination, including palpation of the entire mammary chain to record the number, size, and location of palpable nodules and palpation of inguinal and axillary lymph nodes. Reproductive status (intact *vs* spayed) at the time of surgery was recorded; for dogs with a history of ovariohysterectomy (OHE), the interval between OHE and the index mammary surgery was extracted from the medical record.

Staging routinely included three-view thoracic radiography (right and left lateral plus ventrodorsal or dorsoventral projections) and abdominal ultrasonography to screen for distant metastasis and concurrent disease. Regional lymph nodes (inguinal and axillary) were assessed clinically, and fine-needle aspiration cytology of enlarged or firm nodes was performed at the clinician’s discretion. Clinical staging was assigned using the modified WHO TNM staging framework for canine mammary tumors (39), based on primary tumor size (T), regional lymph node status (N), and the presence or absence of distant metastasis (M); dogs with imaging evidence of distant metastatic disease (M1) were excluded.

### 2.4 Surgical procedures

All surgeries were performed under general anesthesia according to hospital protocols for premedication, induction, maintenance, and intraoperative monitoring. Dogs were positioned in dorsal recumbency, and the thoracoabdominal region was clipped widely and prepared using standard aseptic scrub and draping techniques.

The choice of nodulectomy, regional mastectomy, or unilateral radical mastectomy followed Mammary Oncology Unit guidelines (26) and was based on tumor distribution, size, and local invasiveness. Nodulectomy was reserved for small, well-circumscribed peripheral nodules when adequate lateral and deep margins could be obtained without removing the entire gland. Regional mastectomy consisted of en bloc excision of two or more adjacent glands with associated subcutaneous tissue when nodules were confined to part of the chain or when tumor location precluded nodulectomy. Unilateral radical mastectomy involved en bloc removal of the entire mammary chain on one side, including overlying subcutaneous tissues and, when present, the ipsilateral superficial inguinal lymph node.

Skin incisions were planned to include adequate lateral margins around palpable tumors and any previous surgical scars. Dissection was performed in the subcutaneous plane with meticulous hemostasis. The superficial inguinal lymph node was excised en bloc with the caudal gland when present, whereas axillary lymph nodes were removed when enlarged, firm, or adherent, at the surgeon’s discretion. Deep tissues were closed with absorbable sutures, followed by subcutaneous closure and standard skin closure (intradermal pattern or skin sutures) according to surgeon preference. Procedures were performed by board-certified or nationally accredited soft tissue surgeons, or by residents under direct supervision.

### 2.5 Wound soaker catheter protocol and exposure definition

Wound soaker catheters (WSCs; MILA International®, Florence, KY, United States) were placed intraoperatively as part of multimodal analgesia. After closure of the deep and subcutaneous layers and immediately before skin closure, a multi-perforated catheter was tunneled subcutaneously along the length of the incision. The perforated segment was positioned superficial to the muscular fascia and deep to the skin over the surgical field. The distal end exited through a separate stab incision and was secured to the skin with non-absorbable suture.

Bupivacaine 0.5% was administered through the catheter as intermittent bolus injections at 1–2 mg/kg every 6 h. The intended duration of WSC use was approximately 48 h; in this cohort, the observed median duration was 2 days (interquartile range 2–4 days) (27). Some dogs received all boluses during hospitalization, whereas others were discharged with the catheter in place and owners administered bupivacaine at home according to a written protocol under veterinary supervision. Catheters were removed at a scheduled postoperative recheck according to the institutional protocol. For analysis, WSC exposure was coded as a binary variable (yes/no) based on intraoperative placement, regardless of administration setting or catheter dwell time. The safety and feasibility of this protocol in dogs undergoing mastectomy at the same institution have been reported previously (26, 27).

During the earlier years of the study period, WSC placement was at the discretion of the attending surgeon and was more commonly used in extensive resections. As institutional experience accumulated, WSC use progressively expanded and became more routinely implemented in later years (26, 27). Accordingly, WSC allocation was non-random and related to surgical extent and calendar time, with potential for confounding by indication. This time-dependent implementation implies that the estimated association between WSC use, and local recurrence may partially reflect secular trends in case selection and perioperative/surgical practice and therefore should not be interpreted causally. For the secondary objective, WSC use (yes/no) was defined as the main exposure in relation to time to local recurrence.

### 2.6 Postoperative care, adjuvant treatment, and follow-up

Postoperative management followed Mammary Oncology Unit protocols. Systemic analgesia in the immediate postoperative period followed a multimodal approach that typically included an opioid combined with a non-steroidal anti-inflammatory drug (NSAID) when not contraindicated, with additional adjuncts used at the clinician’s discretion. Perioperative antimicrobial prophylaxis was administered according to institutional guidelines.

Dogs were routinely re-examined 3–5 days and 12–15 days after surgery for postoperative wound evaluation and routine postoperative care. Suture removal was performed as appropriate. Long-term follow-up was scheduled by the Mammary Oncology Unit for up to 2 years, with planned oncology rechecks every 3 months that included physical examination with focused assessment of the surgical site and regional lymph nodes and thoracic radiography at each interval. Abdominal ultrasonography and clinicopathologic testing were performed when clinically indicated, including for dogs receiving adjuvant treatment. Because of the retrospective design, adherence to the scheduled follow-up varied; follow-up information was extracted from documented hospital rechecks and supplemented, when needed, by reports from referring veterinarians and communication with owners. The date of last contact was recorded for time-to-event analyses.

Adjuvant treatment was extracted from the Mammary Oncology Unit medical record and categorized as (1) long-term NSAID therapy (yes/no; agent recorded), defined as NSAID administration beyond the routine immediate postoperative analgesic period, and (2) systemic antineoplastic therapy (yes/no; agent(s) recorded), including cytotoxic chemotherapy and/or tyrosine kinase inhibitors. Because specific regimens were heterogeneous and individual drug categories were infrequent, treatment variables were modeled primarily as binary indicators (long-term NSAID therapy: yes/no; any systemic antineoplastic therapy: yes/no). In an additional coding approach, any adjuvant treatment (long-term NSAID therapy and/or systemic chemotherapy: yes/no) was also evaluated to reduce sparsity and improve interpretability.

### 2.7 Definition of local recurrence, time origin, and censoring

Local recurrence was defined as the development, after the index mammary surgery, of a new mass at or near the prior surgical site within the operated mammary region or adjacent subcutaneous tissues. Only recurrences confirmed histologically as mammary carcinoma on biopsy or excision were considered events. Histopathology reports of suspected recurrences were re-reviewed to confirm that cases met the study definition.

For time to event analyses, time zero was defined as the date of the index mammary surgery, reflecting the study objective and the timing of perioperative exposures, including WSC placement. Time to local recurrence was calculated as the interval (days) between the date of surgery and the date of biopsy/excision of the suspected recurrence (the date tissue was obtained), rather than the date the pathology report was issued. Dogs without histologically confirmed local recurrence were censored at the date of last documented follow-up without recurrence, death without local recurrence, or administrative censoring (December 31, 2024), whichever occurred first.

### 2.8 Variables and data collection

Epidemiologic, clinical, and pathological data were extracted retrospectively from the electronic medical record into a standardized database. For dogs with multiple malignant mammary tumors, the largest tumor diameter was recorded, and histologic variables were assigned to the tumor with the most aggressive histologic features. Tumor-related variables included the number of mammary tumors in the operated chain; maximum tumor diameter (cm), defined as the largest lesion diameter and obtained preferentially from the gross description in the histopathology report (unfixed tissue) when available or otherwise from clinical measurements. Histologic grade (II *vs* III) (7); histologic subtype (37, 38); histologic infiltration (yes/no), defined as neoplastic epithelial cells extending beyond preexisting mammary structures into surrounding tissues, as reported in the final histopathology report; intraoperative evidence of deep tissue adherence (yes/no); ulceration on physical examination (yes/no); and histologic regional lymph node metastasis (yes/no) in inguinal and/or axillary nodes when assessed.

Clinical management variables included the type of surgery (nodulectomy, regional mastectomy, or unilateral radical mastectomy), inguinal lymphadenectomy (yes/no), WSC placement (yes/no), and adjuvant treatment indicators (long-term NSAID therapy and/or any systemic antineoplastic therapy, coded as yes/no as defined above). The primary outcome was time to histologically confirmed local recurrence, as defined above. For descriptive purposes, binary local recurrence status (yes/no) and follow-up time among censored dogs were also derived.

Dogs lacking essential information to determine eligibility, the main exposure (WSC placement), or the outcome were excluded *a priori* during cohort assembly. Remaining missing data were handled by complete-case analysis in regression models; denominators are reported in tables, and the multivariable model was restricted to dogs with complete data for the main predictors.

### 2.9 Statistical analysis

Continuous variables were summarized as medians and interquartile ranges (IQRs), and categorical variables as counts and percentages. For baseline comparisons between dogs with and without WSCs, continuous variables were compared using the Mann–Whitney U test, and categorical variables were compared using Pearson’s chi-squared test or Fisher’s exact test, as appropriate. Local recurrence-free survival (LRFS) was defined as the time from surgery to histologically confirmed local recurrence; dogs without recurrence were censored at last follow-up. LRFS was estimated using the Kaplan–Meier method, and groups were compared using the log-rank test.

“Time to local recurrence was analysed using Cox proportional hazards regression. Univariable Cox models were fitted for candidate predictors (epidemiological, tumour-related and clinical management variables) in order to describe the unadjusted associations. Due to the very small number of outcome events (11 histologically confirmed local recurrences), any multivariable modelling was considered exploratory and intentionally constrained to reduce overfitting and improve interpretability. The final multivariable model was therefore restricted to a parsimonious set of covariates, including the exposure of interest (WSC use, retained *a priori*) and two key clinicopathological predictors selected to represent tumour burden and invasive growth (maximum tumour diameter and histological infiltration). Accordingly, hazard ratio estimates, particularly for WSC use, were expected to be imprecise and the multivariable results are not intended to support causal inference. Calendar period was considered a plausible confounder because WSC uptake increased in later years; however, with only 11 recurrence events and the close correlation between WSC use and calendar time, adding calendar-year/period terms would be expected to produce unstable, overfit estimates. Accordingly, we did not model calendar time as an additional regression covariate, and we interpret the WSC coefficient as exploratory. Follow-up time was summarized using the reverse Kaplan–Meier method, which estimates potential follow-up time by treating censoring as the event. Clinical stage (modified WHO TNM) was recorded for all dogs but was not included as a regression covariate because it is a composite of primary tumor size (T) and regional nodal status (N), which were evaluated directly; given only 11 events, we prioritized modeling tumor size as a continuous predictor and avoided including composite variables that would reduce information and increase collinearity.

Hazard ratios (HRs) with 95% confidence intervals (CIs) were reported. Robust (Huber–White) standard errors were used given the small number of events. The proportional hazards assumption was evaluated using Schoenfeld residuals (covariate-specific and global tests). Model discrimination was assessed with Harrell’s concordance index (C-index). Internal validation of the final multivariable model was performed using nonparametric bootstrap resampling with 3,000 replications to evaluate model stability and potential overfitting. An interaction term between WSC use and histologic infiltration was tested to explore effect modification. Two-sided *p*-values < 0.05 were considered statistically significant; *p*-values are reported to three decimal places except when *p <* 0.001. Analyses were performed using Stata 15.0 (StataCorp LLC, College Station, TX, United States).

## 3 Results

### 3.1 Study population and baseline characteristics

The study included 117 female dogs with histologically confirmed grade II (*n* = 76, 65.0%) or grade III (*n* = 41, 35.0%) mammary carcinomas (Table 1). The median age at surgery was 10.8 years [interquartile range (IQR): 9.0–13.0], and most dogs were intact (*n* = 85, 72.6%), with 32 (27.4%) spayed. The median number of mammary tumors per dog was 2 (IQR: 1–4). Surgical procedures included unilateral radical mastectomy in 74 dogs (63.2%), regional mastectomy in 37 dogs (31.6%), and nodulectomy in 6 dogs (5.1%). Inguinal lymphadenectomy was performed in 92 dogs (78.6%). Histologically confirmed inguinal lymph node metastasis was identified in 27/92 dogs (29.3%). Histological infiltration was reported in 33 dogs (28.2%), and deep tissue adherence at surgery was reported in 29 dogs (24.8%) (Table 1).

**Table 1:** Baseline clinical, tumor-related, and treatment characteristics of 117 female dogs undergoing mammary surgery for histologic grade II–III mammary carcinomas.

Variable	*n* (%) or median (IQR)
Age (years)	10.8 (9.0–13.0)
Body condition score	5.0 (5.0–6.0)
Reproductive status	
Intact	85 (72.6)
Spayed	32 (27.4)
Tumor grade	
Grade II	76 (65.0)
Grade III	41 (35.0)
Number of mammary tumors per dog	2 (1–4)
Maximum tumor diameter (cm)	2.5 (1.0–5.0)*
Type of surgery	
Unilateral radical mastectomy	74 (63.2)
Regional mastectomy	37 (31.6)
Nodulectomy	6 (5.1)
Inguinal lymphadenectomy performed	92 (78.6)
Lymph node metastasis (histologically confirmed)†	27/92 (29.3)
Axillary lymph node not assessed	93 (79.5)
Axillary lymph node metastasis (histologically confirmed)	1/24^‡^ (4.2)
Histologic infiltration	33 (28.2)
Deep tissue adherence	29 (24.8)
Wound soaker catheter (WSC) use	
Yes	67 (57.3)
No	50 (42.7)
Long-term NSAID therapy	
Yes	41 (35.0)
No	76 (65.0)
Systemic chemotherapy (any)	
Yes	24 (20.5)
No	93 (79.5)

Values are presented as *n* (%) or median (IQR). * Maximum tumor diameter was available for 107 dogs. † Inguinal lymph node metastasis was histologically confirmed and is reported as *n*/*N* assessed (%). ^‡^ Axillary lymph node metastasis is reported as n/N assessed (%) because axillary nodes were not assessed in all dogs.

Wound soaker catheters were placed in 67/117 dogs (57.3%). Among dogs with available tumor diameter measurements (45 without WSC; 62 with WSC), maximum tumor diameter was smaller in dogs with WSCs than in dogs without WSCs [median 1.8 cm (IQR: 1.0–4.0) *vs* 3.0 cm (IQR: 1.9–5.5); *p* = 0.041] (Supplementary Figure S1). Unilateral radical mastectomy was more common in the WSC group (74.6% *vs* 48.0%; *p* = 0.006). Other baseline clinicopathologic variables, including histologic infiltration and lymph node metastasis, were similar between groups (Table 2). Histological subtypes and breed distribution are summarized in Supplementary Tables S1, S2.

**Table 2:** Baseline clinicopathologic characteristics of 117 female dogs with histologic grade II–III mammary carcinomas according to wound soaker catheter (WSC) use.

Variable	No WSC (*n* = 50)	WSC (*n* = 67)	*p*-value
Age at surgery, years – median (IQR)	10.5 (8.9–12.1)	11.7 (9.0–13.0)	0.124
Maximum tumor diameter, cm – median (IQR)*	3.0 (1.9–5.5)	1.8 (1.0–4.0)	0.041
Body weight, kg – median (IQR)	13.1 (7.5–20.6)	15.0 (8.2–24.3)	0.428
Number of mammary tumors, median (IQR)	2 (1–4)	2 (1–4)	0.756
Type of surgery, *n* (%)			0.006†
Nodulectomy	5 (10.0)	1 (1.5)	
Regional mastectomy	21 (42.0)	16 (23.9)	
Unilateral radical mastectomy	24 (48.0)	50 (74.6)	
Histologic tumor infiltration, *n* (%)			0.106†
Absent	32 (64.0)	52 (77.6)	
Present	18 (36.0)	15 (22.4)	
Inguinal lymphadenectomy performed, *n* (%)	34 (68.0)	58 (86.6)	0.015†
Inguinal lymph node metastasis (histologically confirmed), *n*/*N* assessed (%)	9/34 (26.5)	18/58 (31.0)	0.643†

IQR, interquartile range; WSC, wound soaker catheter. Values are presented as median (interquartile range, IQR) or *n* (%). Continuous variables were compared using the Mann–Whitney *U*-test. * Maximum tumor diameter data were available for 45 dogs without WSC and 62 dogs with WSC. † Pearson’s chi-squared test.

### 3.2 Local recurrence and follow-up

Histologically confirmed local recurrence was observed in 11/117 dogs (9.4%). The median time to local recurrence among dogs that recurred was 6.4 months (IQR: 3.6–14.1). Median follow-up for the cohort, estimated using the reverse Kaplan–Meier method, was 218 days (95% CI: 192–275). Overall, 35/117 dogs (29.9%) had at least 12 months of follow-up and 15/117 (12.8%) had at least 24 months of follow-up. The proportions with ≥12 months (19/67 *vs* 16/50) and ≥24 months (8/67 *vs* 7/50) of follow-up were similar between dogs with and without WSCs.

**Figure 1:**
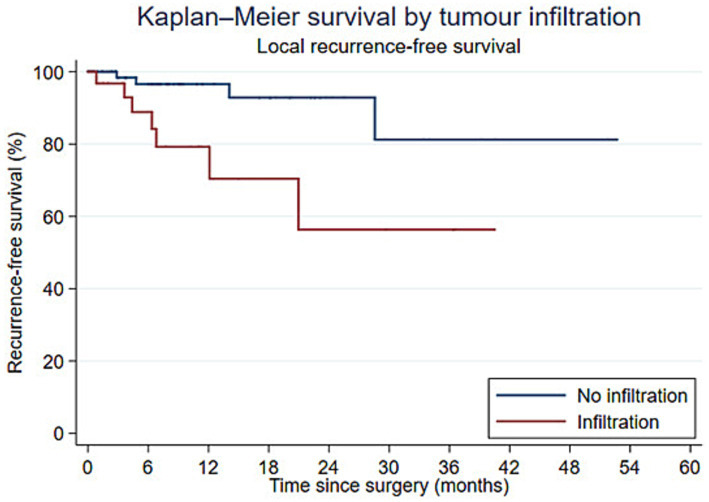
Kaplan–Meier curves for local recurrence-free survival according to histologic infiltration. Dogs with infiltrative tumors had a shorter local recurrence-free survival than those without infiltration (log-rank *p* = 0.020).

**Figure 2:**
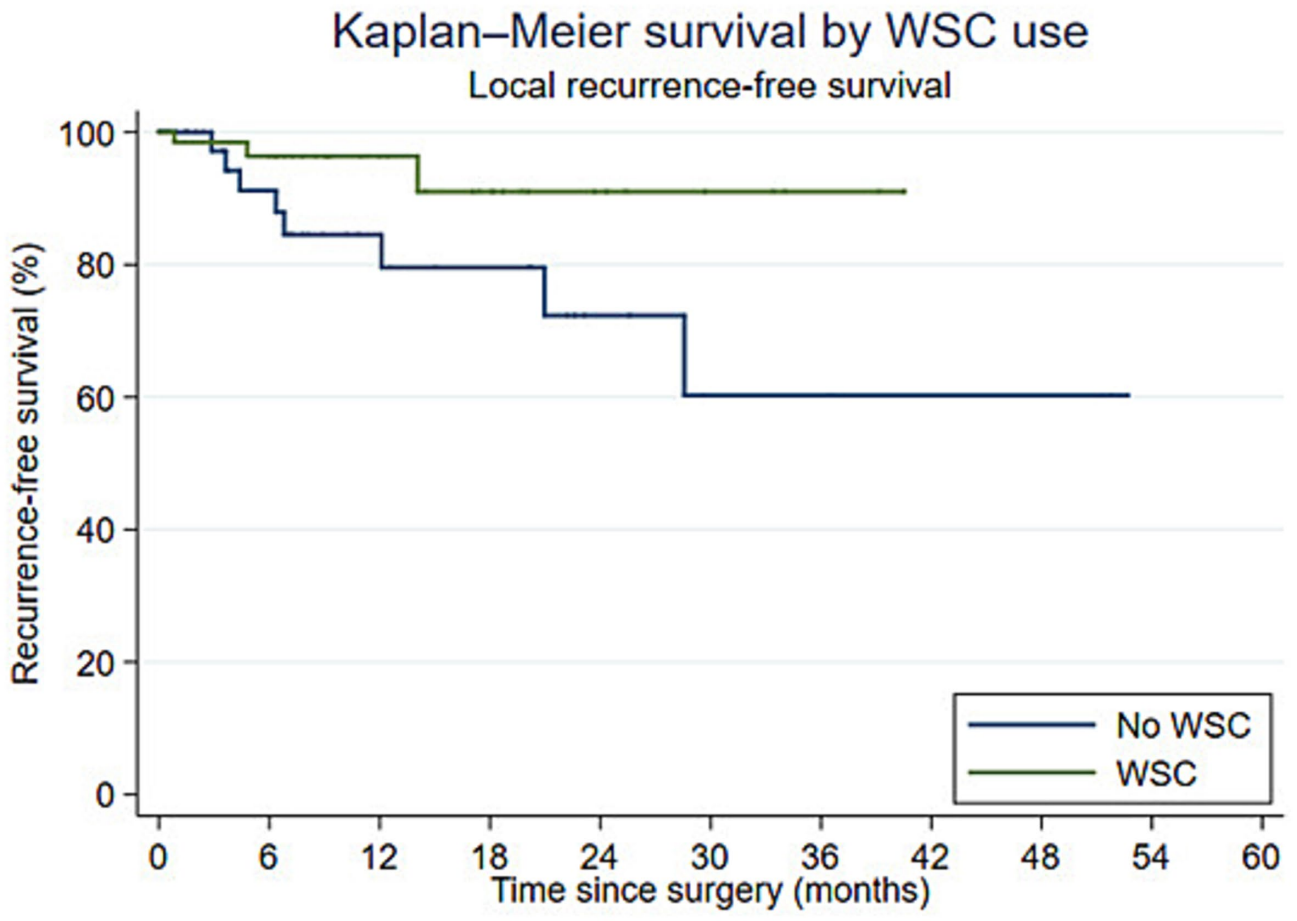
Kaplan–Meier curves for local recurrence-free survival according to wound soaker catheter (WSC) use. Local recurrence-free survival showed no clear evidence of a difference between dogs treated with and without WSCs (log-rank *p* = 0.050).

Kaplan–Meier estimates of local recurrence-free survival differed by histologic infiltration status, with lower survival in dogs with infiltrative tumors (log-rank *p* = 0.020; Figure 1). In contrast, Kaplan–Meier estimates of local recurrence-free survival showed no clear evidence of a difference by WSC use (log-rank *p* = 0.050; Figure 2).

### 3.3 Univariable Cox analyses

Univariable Cox proportional hazards models are presented in Supplementary Table S3. Larger maximum tumor diameter was associated with a higher hazard of local recurrence [hazard ratio (HR): 1.23 per 1-cm increase; 95% confidence interval (CI): 1.05–1.44; *p* = 0.012], and histologic infiltration was also associated with a higher hazard of local recurrence (HR: 4.53; 95% CI: 1.34–15.32; *p* = 0.015). WSC use was not statistically significantly associated with local recurrence in univariable analysis (HR: 0.28; 95% CI: 0.08–1.04; *p* = 0.058). Unilateral radical mastectomy (*vs* nodulectomy or regional mastectomy) was not associated with local recurrence (HR: 1.86; 95% CI: 0.50–6.98; *p* = 0.357). Long-term NSAID therapy (HR: 5.19; 95% CI: 1.17–23.11; *p* = 0.031) and any adjuvant treatment (long-term NSAID therapy and/or systemic chemotherapy; HR: 10.18; 95% CI: 1.31–79.18; *p* = 0.027) were associated with a higher hazard of local recurrence, whereas systemic chemotherapy alone showed a non-significant trend (HR: 2.70; 95% CI: 0.85–8.57; *p* = 0.091). Because these therapies were prescribed based on clinical judgement and tumour characteristics, these univariable associations are likely confounded by indication and should not be interpreted as causal treatment effects. Age, body weight, number of mammary tumors, and inguinal lymph node metastasis were not statistically significant in univariable analyses (all *p* > 0.10; Supplementary Table S3).

### 3.4 Multivariable Cox model

**Figure 3:**
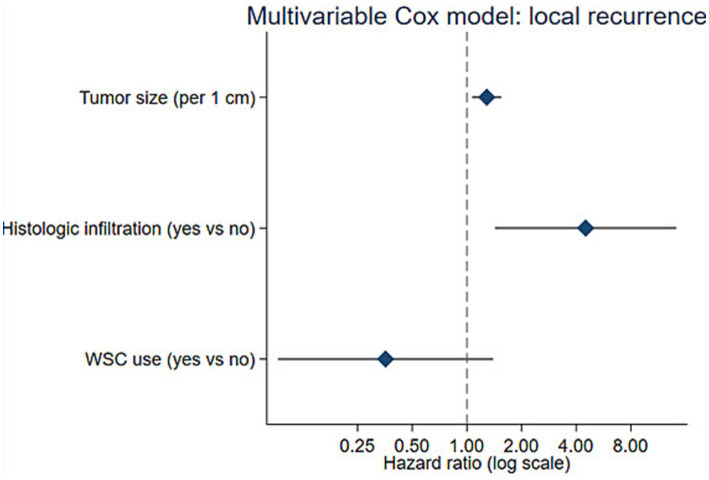
Forest plot of the multivariable Cox proportional hazards model for time to local recurrence. Hazard ratios (HRs) and 95% confidence intervals (CIs) are shown for tumor size (per 1-cm increase), histologic infiltration (present *vs* absent), and wound soaker catheter (WSC) use (yes *vs* no). The vertical dashed line represents the null value (HR = 1.0); HRs are plotted on a logarithmic scale.

Given the limited number of local recurrence events (*n* = 11), multivariable modeling was restricted to a parsimonious set of predictors to limit overfitting. The final Cox proportional hazards model included 107 dogs with complete data for tumor size, histologic infiltration, and WSC use; WSC use was retained *a priori* as the exposure of interest regardless of statistical significance (Table 3; Figure 3). Tumor size and histologic infiltration remained independently associated with local recurrence. Each 1-cm increase in maximum tumor diameter was associated with a higher hazard of local recurrence (HR: 1.29; 95% CI: 1.07–1.55; *p* = 0.008), and dogs with histologic infiltration had a higher hazard of local recurrence than those without infiltration (HR: 4.51; 95% CI: 1.43–14.23; *p* = 0.010). Despite restricting the model to three covariates, the low event count implies an increased risk of overfitting and unstable effect estimates; therefore, the multivariable hazard ratios should be interpreted cautiously as exploratory and hypothesis-generating rather than confirmatory.

**Table 3:** Multivariable Cox proportional hazards model for time to local recurrence in 107 female dogs with histologic grade II–III mammary carcinomas.

Variable	Hazard ratio (HR)	95% CI	*p*-value
Tumor size (per cm increase)	1.29	1.07–1.55	0.008
Histologic infiltration (present)	4.51	1.43–14.23	0.010
Wound soaker catheter (yes)	0.36	0.09–1.40	0.138

HR, hazard ratio; CI, confidence interval; WSC, wound soaker catheter. Harrell’s C-index = 0.80. The model was internally validated using 3,000 bootstrap replications.

After adjustment for tumor size and histologic infiltration, WSC use was not statistically significantly associated with local recurrence (HR: 0.36; 95% CI: 0.09–1.40; *p* = 0.138). Long-term NSAID therapy and systemic chemotherapy were not retained in the final model to preserve parsimony given the small number of events.

### 3.5 Model performance and assumption checks

The multivariable Cox model showed good discrimination, with a Harrell’s C-index of 0.80 (Table 3). Internal validation by nonparametric bootstrap resampling with 3,000 replications yielded similar effect estimates, standard errors, and confidence intervals for all covariates (Supplementary Table S4). No relevant deviations from the proportional hazards assumption were detected based on Schoenfeld residuals. No statistically significant interaction between WSC use and histologic infiltration was identified.

## 4 Discussion

This retrospective cohort study evaluated clinicopathologic predictors of histologically confirmed local recurrence after mammary surgery in female dogs with grade II-III mammary carcinomas and assessed whether wound soaker catheters (WSCs) delivering intermittent bupivacaine boluses were associated with recurrence hazard. A retrospective design was a pragmatic approach for this outcome, which was uncommon in this cohort (11 events) and would require large prospective accrual and prolonged follow-up to study efficiently. In the parsimonious multivariable Cox model, maximum tumor diameter (HR: 1.29 per 1-cm increase; 95% CI: 1.07–1.55) and histologic infiltration (HR: 4.51; 95% CI: 1.43–14.23) were independently associated with a higher hazard of local recurrence, whereas WSC use was not (HR: 0.36; 95% CI: 0.09–1.40). The WSC estimate was imprecise and did not support either increased recurrence hazard or a protective effect within the limits of a small event count.

The univariable associations observed for long-term NSAID therapy and for the composite indicator of any adjuvant treatment warrant cautious interpretation. In this retrospective cohort, adjuvant approaches were not assigned randomly; rather, they were prescribed based on clinician judgement and patient- and tumour-related characteristics. Consequently, these treatment variables likely function as markers of underlying disease severity and case selection rather than as independent causal determinants of local recurrence. Importantly, our findings should not be interpreted as evidence that NSAIDs or systemic antineoplastic therapy increase recurrence risk.

Considering that only 11 histologically confirmed local recurrences occurred, all Cox regression findings, especially the WSC estimate, should be viewed as exploratory; wide confidence intervals reflect limited precision, and the analysis is not suitable for causal interpretation. The association between tumor size and local recurrence is consistent with prior canine mammary tumor studies in which larger lesions are linked to poorer outcomes, including shorter disease-free and overall survival (1, 2, 40). Peña *et al*. (2013) and Rasotto *et al*. (2017) reported tumor diameter as an independent prognostic factor, with increasing risks beyond commonly used thresholds such as 3–5 cm (7, 8). Our findings extend these observations by focusing on a recurrence-specific, histologically confirmed endpoint and by restricting the analysis to grade II-III carcinomas managed under a standardized mammary oncology protocol (7, 13). Within this relatively homogeneous group of intermediate- and high-grade tumors, tumor size remained a key correlate of local control.

Local invasiveness is also widely recognized as an adverse prognostic factor in canine mammary carcinoma. Invasive growth patterns, lymphovascular invasion, and incomplete or close surgical margins have been associated with increased local recurrence and reduced survival (8, 38, 40, 41). In this cohort, histologic infiltration, defined as neoplastic epithelial cells extending beyond preexisting mammary structures into surrounding tissues, was independently associated with local recurrence. Clinical stage (modified WHO TNM) was recorded, but because it is a composite of primary tumor extent (T) and nodal status (N), tumor size was modeled directly as a continuous predictor rather than including stage as a covariate in a dataset with only 11 events. Margin status/width and lymphovascular invasion were not consistently retrievable from historical pathology reports and therefore could not be evaluated as predictors; in this context, histologic infiltration provides a pragmatic indicator of invasive growth. The lack of an independent association between histologic grade and time to local recurrence likely reflects the intentionally restricted inclusion of grades II and III and the limited number of local recurrence events, whereas a clearer prognostic gradient is more consistently reported in cohorts including grades I-III and focusing on broader outcomes rather than isolated local recurrence (7–9).

The proportion of local recurrences observed here (9.4%) is at the lower end of the range reported for malignant mammary tumors in dogs (1, 40, 42). Several cohort features may contribute. All dogs were managed in a single Mammary Oncology Unit under a long-standing protocol that standardizes staging, surgical planning, histopathology reporting, analgesic management, and follow-up (7, 13). In addition, local recurrence required histological confirmation, reducing misclassification with *de novo* tumors. However, follow-up was heterogeneous and overall modest (reverse Kaplan–Meier median 218 days), with fewer than one-third of dogs observed for ≥12 months and only 15/117 (12.8%) observed for ≥24 months. Therefore, late local recurrences may have been missed, and the cumulative incidence of local failure may be underestimated; accordingly, the present findings are most relevant to early postoperative local recurrence. With respect to WSCs, recurrence-focused clinical evidence on their oncologic impact in dogs undergoing mammary surgery remains limited. In this cohort, after adjustment for tumor size and histologic infiltration, the use of a subcutaneous WSC to deliver intermittent bupivacaine boluses for approximately 48 h postoperatively was not associated with a higher hazard of local recurrence. The point estimate was below 1, but the confidence interval was wide and compatible with modest harmful, neutral, or beneficial effects; therefore, these data should not be interpreted as evidence of a protective effect. Most veterinary studies on WSCs have focused on analgesic and wound outcomes rather than tumor recurrence; overall, WSCs are feasible and have been associated with low complication rates and no apparent increase in surgical-site infection when placed and managed under strict aseptic conditions (21, 22, 24, 43). Recent reports in dogs undergoing mastectomy, including a small case series from the same institution and a larger cohort evaluating surgical-site infection, similarly did not identify increased infection rates associated with wound catheters (26, 27). Taken together, our findings provide no evidence of increased local recurrence hazard associated with WSC use within the available follow-up, while emphasizing the need for larger cohorts to estimate modest effects with greater precision. Importantly, WSC placement in this cohort was not randomized and expanded in later calendar years. Baseline imbalances between dogs with and without WSCs (Table 2), including smaller tumors and numerically less frequent infiltrative growth in the WSC group, but more frequent unilateral radical mastectomy and inguinal lymphadenectomy, illustrate that confounding by indication and secular changes in practice may operate in opposing directions. Consequently, the observed WSC hazard ratio should be interpreted as hypothesis-generating and cannot be used to infer a causal (protective or harmful) effect, nor as definitive evidence of oncological safety.

The long-standing surgical oncology concern that devices traversing a tumor bed could provide a route for tumor-cell implantation has historically led to caution regarding drains and catheters (28, 30). In human breast cancer, isolated case reports describe drain-tract metastasis (29), and recommendations emphasize careful placement such that tracts can be encompassed within subsequent treatment fields if needed. These events appear rare, and there is no established clinical evidence that wound infusion catheters used for analgesia increase recurrence rates in human or veterinary oncology. The present findings are consistent with this neutral clinical picture: within a standardized care pathway. Within this cohort, we did not detect a higher hazard of local tumor regrowth associated with WSC placement; however, this should not be interpreted as definitive evidence of oncologic safety.

Whether anesthetic and analgesic techniques influence cancer outcomes has attracted considerable interest in human medicine. Early retrospective studies and meta-analyses suggested that regional anesthesia might be associated with lower recurrence or metastasis, potentially through attenuation of the surgical stress response, opioid-sparing effects, and systemic actions of local anesthetics (33, 44). However, randomized evidence in women undergoing breast cancer surgery did not detect differences in cancer recurrence between regional anesthesia–analgesia and general anesthesia approaches (34). In parallel, experimental studies indicate that bupivacaine and other local anesthetics can induce apoptosis and reduce proliferation and migration in tumor models, including a canine mammary tumor cell line (32), and can reduce tumor growth in murine models (45). Proposed mechanisms include voltage-gated sodium channel–related effects and sodium channel-independent pathways involving intracellular signaling (Akt/mTOR) and mitochondrial bioenergetics (31, 46). Overall, clinical data do not indicate higher recurrence rates attributable to locoregional techniques, while laboratory studies provide biologic plausibility for antitumor effects of local anesthetics; the clinical relevance of these effects remains uncertain (35). Any potential antitumor contribution of bupivacaine delivered via WSCs in dogs therefore remains speculative and cannot be inferred from the present study.

This study has several strengths. The cohort was clinically well characterized and restricted to female dogs with histologically confirmed grade II or III mammary carcinomas and no evidence of distant metastasis at diagnosis (M0), managed at a single teaching hospital within a dedicated Mammary Oncology Unit under a long-standing protocol. Local recurrence was defined strictly as histologically confirmed regrowth at or near the surgical site, improving endpoint specificity. Finally, the time-to-event approach (Cox proportional hazards with robust standard errors), proportional hazards diagnostics, assessment of discrimination, and internal bootstrap validation support the internal consistency of the main associations observed for tumor size and histologic infiltration.

Several limitations should be acknowledged. First, the number of outcome events very small (11 histologically confirmed local recurrences). Although we deliberately limited multivariable adjustment to a parsimonious model and explored stability using bootstrap resampling, the events-per-parameter ratio remains low and hazard ratio estimates may be unstable. Consequently, these results should be interpreted as hypothesis-generating and cannot support causal conclusions regarding the independent effects of WSC use or other covariates. Follow-up was heterogeneous and overall modest (reverse Kaplan–Meier median 218 days), and only 15/117 (12.8%) dogs were observed for ≥24 months; therefore, late local recurrences may have been missed and cumulative local failure may be underestimated. Accordingly, these analyses primarily inform early local recurrence and should not be extrapolated to long-term local control. Requiring histologic confirmation improves specificity but may miss clinically suspected recurrences that were not sampled. Because WSC placement was nonrandomized and adopted over time, its association with local recurrence may be confounded by temporal changes in practice and residual confounding by indication despite adjustment for major tumor characteristics. In addition, interpretation of hazard ratios for long-term NSAID therapy and systemic chemotherapy is limited by strong confounding by indication and disease severity, and these estimates should not be interpreted as evidence of treatment-related harm. Finally, margin status/width and lymph vascular invasion were not consistently retrievable from historical pathology reports, precluding their evaluation as predictors and limiting comparability with cohorts incorporating standardized margin assessment; histologic infiltration was therefore used as a pragmatic indicator of local invasiveness.

From a clinical standpoint, these findings underscore that tumor burden and invasive growth features remain key correlates of local recurrence after mammary surgery for intermediate and high-grade canine mammary carcinomas. Large and infiltrative tumors should be recognized as higher-risk lesions, supporting careful preoperative planning to achieve adequate lateral and deep margins when anatomically feasible and closer postoperative surveillance for early detection of local regrowth. Within the available follow-up, WSC-based local analgesia may be considered as part of multimodal perioperative pain management when clinically indicated; however, our data should be interpreted as exploratory and cannot be taken as definitive evidence of oncologic safety. Future prospective cohorts with standardized and sufficiently long follow-up are needed to refine risk stratification for local recurrence and to estimate the WSC-recurrence association with greater precision. Where feasible, integrating molecular tumor characterization may help identify biologic subtypes with distinct local behavior and clarify whether tumor biology modifies associations between invasiveness, surgical variables, and postoperative analgesic strategies, while keeping the primary focus on clinically meaningful, recurrence-specific endpoints.

## 5 Conclusion

In this retrospective cohort of 117 female dogs with histologic grade II or III mammary carcinomas treated surgically, maximum tumor diameter and histologic infiltration were independently associated with a higher hazard of histologically confirmed local recurrence. Wound soaker catheter use delivering intermittent bupivacaine boluses was not associated with a higher hazard of local tumor regrowth within the available follow-up; however, the estimate was imprecise and, given the observational design and low event count, these findings should be interpreted as exploratory and not as definitive evidence of oncologic safety. Median potential follow-up was 218 days and long-term follow-up was limited; therefore, these findings primarily pertain to early local recurrence and do not exclude late local failures. Larger cohorts with longer standardized follow-up are warranted to improve precision and better exclude modest effects.

## Data Availability

The raw data supporting the conclusions of this article will be made available by the authors, without undue reservation.
