# Supplementary material for: Predictors of histologically confirmed local recurrence and no evident association of wound soaker catheter use with local recurrence in dogs with grade II–III mammary carcinomas: a retrospective cohort study
Source: Front Vet Sci. 2026 Mar 27;13:1772385. doi: 10.3389/fvets.2026.1772385 (PMC13067888; doi:10.3389/fvets.2026.1772385)

Supplementary Material

**Supplementary Table 1.** Histologic classification of malignant mammary tumors in 117 female dogs.

| **Histologic subtype** | **n** | % |
| --- | --- | --- |
| Solid carcinoma | 24 | 20.5 |
| Tubular carcinoma | 17 | 14.5 |
| Comedocarcinoma | 15 | 12.8 |
| Complex carcinoma | 13 | 11.1 |
| Tubulopapillary carcinoma | 11 | 9.4 |
| Ductal carcinoma | 11 | 9.4 |
| Mixed carcinoma | 6 | 5.1 |
| Adenosquamous carcinoma | 6 | 5.1 |
| Anaplastic carcinoma | 3 | 2.6 |
| Carcinoma and malignant myoepithelioma | 3 | 2.6 |
| Cystic-papillary carcinoma | 2 | 1.7 |
| Carcinoma arising in complex adenoma | 2 | 1.7 |
| Intraductal papillary carcinoma | 2 | 1.7 |
| Squamous cell carcinoma | 1 | 0.9 |
| Invasive micropapillary carcinoma | 1 | 0.9 |

**Supplementary Table 2.** Breed distribution of 117 female dogs included in the study.

| **Breed** | **n** | **%** |
| --- | --- | --- |
| Crossbreed | 44 | 37.6 |
| Cocker Spaniel | 9 | 7.7 |
| German Shepherd | 9 | 7.7 |
| Beagle | 6 | 5.1 |
| French Bulldog | 5 | 4.3 |
| Yorkshire Terrier | 5 | 4.3 |
| Poodle | 4 | 3.4 |
| Dachshund | 3 | 2.6 |
| Boxer | 3 | 2.6 |
| German Shorthaired Pointer | 2 | 1.7 |
| Bull Terrier | 2 | 1.7 |
| Golden Retriever | 2 | 1.7 |
| Maltese | 2 | 1.7 |
| Miniature Schnauzer | 2 | 1.7 |
| Pit Bull | 2 | 1.7 |
| West Highland White Terrier | 2 | 1.7 |
| Bichon Frisé | 1 | 0.9 |
| Old English Sheepdog | 1 | 0.9 |
| Brittany Spaniel | 1 | 0.9 |
| Cairn Terrier | 1 | 0.9 |
| Catalan Sheepdog | 1 | 0.9 |
| Cavalier King Charles Spaniel | 1 | 0.9 |
| Chihuahua | 1 | 0.9 |
| English Bulldog | 1 | 0.9 |
| Irish Terrier | 1 | 0.9 |
| Labrador Retriever | 1 | 0.9 |
| Navarra Pointer (Pachón Navarro) | 1 | 0.9 |
| Pekingese | 1 | 0.9 |
| Shih Tzu | 1 | 0.9 |
| Siberian Husky | 1 | 0.9 |
| Staffordshire Bull Terrier | 1 | 0.9 |

**Supplementary Table 3.** Univariable Cox proportional hazards models for time to local recurrence in 117 female dogs with histologic grade II–III mammary carcinomas (events = 11).

| **Variable** | **Coding / unit** | **HR (95% CI)** | **P-value** |
| --- | --- | --- | --- |
| Age | Per year | 1.01 (0.80–1.27) | 0.923 |
| Body weight | Per kg | 0.94 (0.87–1.03) | 0.187 |
| Ovariohysterectomy (OHE) | Yes vs no | 0.46 (0.14–1.57) | 0.218 |
| Days from OHE to surgery | Per day | 1.00 (1.00–1.00)* | 0.243 |
| Number of mammary tumors | Per tumor | 1.10 (0.85–1.43) | 0.457 |
| Maximum tumor diameter | Per cm increase† | 1.23 (1.05–1.44) | 0.012 |
| Unilateral radical mastectomy | Yes vs no | 1.86 (0.50–6.98) | 0.357 |
| Inguinal lymph node metastasis | Yes vs no | 2.18 (0.63–7.48) | 0.216 |
| Histologic infiltration | Yes vs no | 4.53 (1.34–15.32) | 0.015 |
| WSC use | Yes vs no | 0.28 (0.08–1.04) | 0.058 |
| Deep tissue adherence | Yes vs no | 2.35 (0.70–7.90) | 0.167 |
| Ulceration | Yes vs no | 2.28 (0.49–10.51) | 0.291 |
| Inguinal lymphadenectomy performed | Yes vs no | 0.74 (0.19–2.88) | 0.664 |
| Long-term NSAID therapyϮ | Yes vs no | 5.19 (1.17–23.11) | 0.031 |
| Systemic chemotherapy (any) | Yes vs no | 2.70 (0.85–8.57) | 0.091 |
| Any adjuvant treatment (NSAID and/or systemic) | Yes vs no | 10.18 (1.31–79.18) | 0.027 |

*Abbreviations: HR, hazard ratio; CI, confidence interval; WSC, wound soaker catheter; NSAID, non-steroidal anti-inflammatory drug; OHE, ovariohysterectomy. *Modeled per day; the HR rounds to 1.00 because of the unit scaling. †Tumor diameter was available for 107 dogs. Ϯ Long-term NSAID therapy and adjuvant treatment are highly susceptible to confounding by indication and disease severity and should not be interpreted as causal treatment effects Long-term NSAID therapy denotes NSAID use beyond standard short-course postoperative analgesia.*

**Supplementary Table 4.** Bootstrap resampling assessment of model stability of the multivariable Cox proportional hazards model for time to local recurrence.

| **Variable** | **HR (observed)** | **Bootstrap SE** | | **95% CI (bootstrap)** | **Bootstrap P-value** |
| --- | --- | --- | --- | --- | --- |
| Tumor size (per cm increase) | 1.29 | | 0.13 | 1.07–1.55 | 0.008 |
| Histologic infiltration (present vs absent) | 4.51 | | 2.64 | 1.43–14.23 | 0.010 |
| WSC use (yes vs no) | 0.36 | | 0.25 | 0.09–1.40 | 0.138 |

*Bootstrap estimates were obtained using 3,000 replications to assess the internal stability of the multivariable Cox proportional hazards model, which included tumor size (continuous), histologic infiltration (present vs absent) and wound soaker catheter (WSC) use (yes vs no). HR, hazard ratio; SE, standard error; CI, confidence interval; WSC, wound soaker catheter. Bootstrap estimates were obtained using 3,000 replications to explore model stability and potential overfitting. The bootstrap SE is reported on the HR scale. The results are descriptive, not confirmatory.*

**Supplementary Figure S1.** Distribution of maximum tumor diameter in 117 female dogs with histologic grade II–III mammary carcinomas. Histogram and kernel density plot illustrating the right-skewed distribution of maximum tumor diameter. Histogram and kernel density plot illustrating the right-skewed distribution of maximum tumor diameter; most tumors measured between 1 and 4 cm, with a smaller proportion of larger lesions.


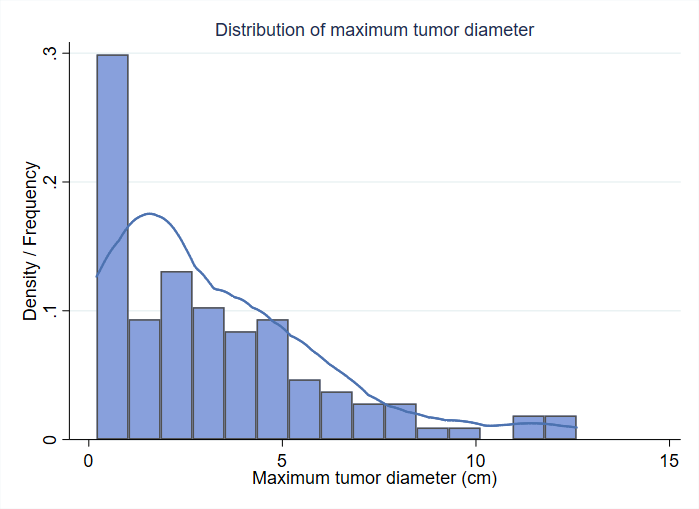

Supplement: Supplementary file 1 [file Table_1.DOCX]
